# DNA Blocks the Lethal Effect of Human Beta-Defensin 2 Against *Neisseria meningitidis*

**DOI:** 10.3389/fmicb.2021.697232

**Published:** 2021-06-30

**Authors:** Gabriela M. Wassing, Kenny Lidberg, Sara Sigurlásdóttir, Jonas Frey, Kristen Schroeder, Nathalie Ilehag, Ann-Christin Lindås, Kristina Jonas, Ann-Beth Jonsson

**Affiliations:** ^1^Department of Molecular Biosciences, The Wenner-Gren Institute, Stockholm University, Stockholm, Sweden; ^2^Science for Life Laboratory, Department of Molecular Biosciences, The Wenner-Gren Institute, Stockholm University, Stockholm, Sweden

**Keywords:** *Neisseria meningitidis*, infection, hBD2, aggregation, eDNA

## Abstract

*Neisseria meningitidis* is a gram-negative bacterium that often asymptomatically colonizes the human nasopharyngeal tract. These bacteria cross the epithelial barrier can cause life-threatening sepsis and/or meningitis. Antimicrobial peptides are one of the first lines of defense against invading bacterial pathogens. Human beta-defensin 2 (hBD2) is an antimicrobial peptide with broad antibacterial activity, although its mechanism of action is poorly understood. Here, we investigated the effect of hBD2 on *N. meningitidis*. We showed that hBD2 binds to and kills actively growing meningococcal cells. The lethal effect was evident after 2 h incubation with the peptide, which suggests a slow killing mechanism. Further, the membrane integrity was not changed during hBD2 treatment. Incubation with lethal doses of hBD2 decreased the presence of diplococci; the number and size of bacterial microcolonies/aggregates remained constant, indicating that planktonic bacteria may be more susceptible to the peptide. Meningococcal DNA bound hBD2 in mobility shift assays and inhibited the lethal effect of hBD2 in a dose-dependent manner both in suspension and biofilms, supporting the interaction between hBD2 and DNA. Taken together, the ability of meningococcal DNA to bind hBD2 opens the possibility that extracellular DNA due to bacterial lysis may be a means of *N. meningitidis* to evade immune defenses.

## Introduction

*Neisseria meningitidis* is a strictly human pathogen that inhabits the nasopharynx asymptomatically in approximately 10% of the population (Christensen et al., 2010). Occasionally, bacteria cross the epithelial and endothelial barrier where they can establish an infection and cause life-threatening sepsis and/or meningitis (van Deuren et al., 2000; Virji, 2009; Pace and Pollard, 2012). The first step toward meningococcal pathogenesis is attachment to host epithelial cells, which is mediated by type IV pili (tfp). These long, filamentous structures protrude from the bacterial surface and are associated with aggregation, adhesion, motility and DNA uptake (Wolfgang et al., 1998; Morand, 2004). After initial adhesion to the epithelium, bacteria form three-dimensional aggregates termed microcolonies (Pujol et al., 1999), which can develop into biofilms that are better protected against environmental conditions (Arenas and Tommassen, 2017). The polysaccharide capsule of *N. meningitidis* mediates resistance against complement-mediated killing and the inhibition of phagocytosis and intracellular killing (Spinosa et al., 2007).

Antimicrobial peptides (AMPs) are highly conserved molecules that are found in mammals, insects and plants. They are relatively small peptides that are often positively charged and amphipathic in nature. They constitute the first line of defense against invading pathogens and contribute to eliminating infection by direct killing of microbes (Zasloff, 2002). Bacterial killing can proceed by membrane disruptive and/or non-membrane disruptive mechanisms (Brogden, 2005). Initial interaction occurs through electrostatic attraction, where positively charged AMPs bind to negatively charged bacterial surfaces. The attached peptides aggregate and insert into the bacterial membrane bilayer, aligning the hydrophobic and hydrophilic portions of the peptide with those of the bacterial lipid membrane. Membrane disruptive killing results in pore formation, destruction of the transmembrane potential and consequently inhibition of respiration, which leads to lysis. Killing through a non-membrane disruptive mechanism occurs when AMPs enter the cell through transient pore formation or through uptake by peptide transporters (Mattiuzzo et al., 2007) and subsequently target intracellular components that can inhibit DNA or protein synthesis or processes such as chaperone-assisted protein folding, enzymatic activity or cell wall formation (Brogden, 2005).

Human beta-defensin 2 (hBD2) is an AMP belonging to the defensin family. Defensins are characterized by a beta sheet structure stabilized by three disulfide bridges (Selsted et al., 1993) and are expressed on epithelial surfaces throughout the human body (O’Neil et al., 1999; Ali et al., 2001; Harder et al., 2001). It is known that hBD2 expression is regulated in response to bacteria or bacterial factors and immune modulators (Chadebech et al., 2003; Tsutsumi-Ishii and Nagaoka, 2003; Wassing et al., 2015). hBD2 exhibits killing activity against various bacterial species, including *Escherichia coli*, *Porphyromonas gingivalis*, and *Pseudomonas aeruginosa* (Singh et al., 1998; Joly et al., 2004). While there is evidence that hBD2 permeabilizes the bacterial membrane (Hoover et al., 2000; Mathew and Nagaraj, 2017), the complete mechanism of action remains poorly understood.

In this work, we investigated the effect of hBD2 on *N. meningitidis*. We found that hBD2 binds to and kills *N. meningitidis* in a time-dependent manner. Planktonic bacteria disappeared upon hBD2 treatment, suggesting that bacterial aggregates are better protected against hBD2 killing. Furthermore, we found that meningococcal DNA binds to hBD2 and that the presence of DNA protects against the peptide, suggesting a potential evasion strategy employed by *N. meningitidis* against hBD2-mediated killing both in suspension and in biofilms.

## Materials and Methods

### Bacterial Strains and Cell Lines

*Neisseria meningitidis* serogroup C strain FAM20 and its *pilE* mutant (Δ*pilE*) have been previously described (Rahman et al., 1997; Jones et al., 2009; Engman et al., 2016). The strains were grown on GC agar (Acumedia) with 1% Kellogg’s supplements (Kellogg et al., 1968) at 37°C and 5% CO_2_ for 16-18 h before experiments. *Lactobacillus reuteri* ATCC PTA5289 (de Klerk et al., 2016) was grown at 37°C 5% CO_2_ on Rogosa (Oxoid) agar for 30-72 h and then in MRS broth for 16-18 h. *Escherichia coli* DH5α was grown on LA-plates and then in LB broth (Acumedia) at 37°C 5% CO_2_ at 200 rpm shaking.

The human pharyngeal epithelial cell line FaDu (ATCC-HBT43) was cultured in Dulbecco’s modified Eagle’s medium (DMEM) (Thermo Fisher Scientific, GibcoTM) and supplemented with 10% heat-inactivated fetal bovine serum (FBS) (Sigma-Aldrich) at 37°C and 5% CO_2_ in a humidified environment.

### Synthetic hBD2

Synthetic hBD2 peptide (GIG DPV TCL KSG AIC HPV FCP RRY KQI GTC GLP GTK CCK KP) was synthesized by Innovagen, Lund, Sweden. hBD2 was dissolved in sterile water at a concentration of 0.4 mg/ml and stored at −80°C.

### Killing Assays

Bacteria from plates were grown to log phase in GC broth supplemented with 1% Kellogg’s at 37°C and 5% CO_2_ for 2 h under shaking conditions. Log phase bacteria were suspended to 10^6^ CFU/ml in modified Dulbecco’s Modified Eagle’s Medium (DMEM, Thermo Fisher Scientific), i.e., DMEM diluted 4× in sterile water, and then mixed with hBD2. It is known that the killing capacity of hBD2 is reduced in the presence of serum and high salt concentration (Bals et al., 1998; Harder et al., 2001; Vylkova et al., 2007); for this reason, we used a diluted form of cell culture medium to perform our killing assay in order to observe an effect by hBD2 while maintaining a physiologically relevant environment. For experiments using bacteria collected directly from plates, the bacteria were suspended in modified DMEM to 10^6^ CFU/ml. Fifty microliters of bacterial suspension was mixed with 10 μl of hBD2 (final concentration of 0.1, 0.5, 1, 5, or 15 μM) and incubated in polypropylene tubes for 1, 2, 3, or 6 h at 37°C and 5% CO_2_ under shaking conditions. Sterile water instead of hBD2 served as the control. Bacterial viability was assessed by serial dilutions, which were plated for viable counts and enumerated the next day. Samples were carefully vortexed and pipetted up and down and examined by microscopy before plating to check for absence of aggregates.

### ELISA-hBD2 Binding Assay

Bacteria from plates were grown to log phase, diluted to 10^6^ CFU/ml, and incubated as in the killing assays above. For the binding assay, bacteria were incubated in either 0.1 μM or 1 μM hBD2 for 3 h. Samples were washed once in PBS and resuspended in PBS. Bacteria-bound hBD2 was detected using an hBD2 ELISA development kit (Peprotech) according to the manufacturer’s instructions.

### 1-N-Phenylnaphthylamine Outer Membrane Permeability Assay

To measure hBD2-induced permeabilization of the outer membrane, we used a previously described protocol (Alakomi et al., 2000). This method is based on the uptake of 1-N-phenylnaphthylamine (NPN) by bacterial membranes. NPN is a hydrophobic dye whose fluorescence is greatly enhanced in hydrophobic environments; an increase in fluorescence thus indicates a breach in the outer membrane (Trauble and Overath, 1973). A 0.5 mM stock solution of NPN (Sigma-Aldrich) was prepared in acetone and diluted to 40 μM in 5 mM HEPES (Thermo Fisher Scientific) to generate the NPN buffer. Wells of a black-walled, clear bottom microplate (Greiner Bio-one) were filled with 25 μl of NPN-buffer (final concentration, 10 μM) and 25 μl of hBD2 (final concentration, 0.1 μM or 1 μM) or lactic acid (final concentration, 10 mM). Log-phase bacteria were centrifuged at 5000 × *g* for 3 min and resuspended in 5 mM HEPES buffer to 10^7^ CFU/ml, and 50 μl of the bacterial suspension was added to the microplate. The plate was incubated at 37°C, and the fluorescence was immediately measured every minute for 3 h using a Spectramax i3x microplate reader (Molecular Devices) with an excitation wavelength of 485 nm and emission wavelength of 535 nm.

### Flow Cytometry

Bacteria from plates were grown to log phase, diluted to 10^6^ CFU/ml, and incubated as in the killing assays above. For flow cytometry, bacteria were incubated in either 0.1 μM hBD2, 1 μM hBD2, 2 μg/ml tetracycline or 10 μg/ml cephalexin for 2 h. Samples were washed once in PBS before fixation in 4% formaldehyde solution. Cells were permeabilized with 0.1% Triton X-100 and resuspended in 50 mM sodium citrate buffer. Samples were stained with 2.5 μM SYTOX Green (Thermo Fisher Scientific) before being analyzed by flow cytometry using an LSRFortessa flow cytometer (BD Biosciences). For each sample, 30 000 cells were counted. Data were analyzed with FlowJo software.

### Fluorescence Microscopy and Aggregate Quantification

Bacteria from plates were grown to log phase, diluted to 10^6^ CFU/ml, and incubated as in the killing assays above. For microscopy, bacteria were incubated in 0.1 μM hBD2, 1 μM hBD2, or 10 μg/ml cephalexin for 2 h. Samples were washed once in PBS before fixing in 4% formaldehyde solution. Cells were permeabilized with 0.1% Triton X-100 and resuspended in PBS supplemented with 0.2% Tween-20. Cells were stained with 20 nM DAPI for 30 min and resuspended in PBS. A small volume of cells was mounted on 1% agarose pads and sealed under a coverslip. Images were obtained using a T*i* eclipse inverted research microscope with a 100×/1.45 NA objective (Nikon). Fluorescence images were captured using excitation and emission filter cubes for Hoechst 33258. Image preparation was performed using Fiji software. The assay was performed two times with at least 12 images captured per sample. Aggregates were enumerated by counting and were defined as a cluster consisting of more than 4 cells to exclude the possibility of diplococci undergoing division. The relative number of aggregates was calculated as the number of aggregates divided by the number of diplococci.

### Spectrophotometric Quantification of Bacterial Aggregation

To quantify aggregation of the bacterial strains, we used a previously described sedimentation assay (Helaine et al., 2005) with slight modifications. Bacteria were resuspended in supplemented GC broth and filtered through 5 μm filters (Filtropur) to remove aggregates. Bacterial suspensions were adjusted to an OD_600_ of 0.1 and incubated for 3 h at 37°C and 5% CO_2_ under shaking conditions. Bacterial cultures were centrifuged at 2500 × *g* for 5 min, and the pellet was resuspended in an equal volume of the modified DMEM used for the killing assays. Bacterial suspensions were incubated at room temperature under static conditions, and the OD_600_ of the supernatant was measured at 20 min intervals. Absorbance values were normalized against the first time point (0 min).

### DNA Electrophoretic Mobility Shift Assay

Bacterial genomic DNA (gDNA) was isolated using the Wizard Genomic DNA Purification kit (Promega) according to the manufacturer’s instructions. PCR fragments were amplified with Phusion DNA polymerase (Thermo Fisher Scientific). For *N. meningitidis* FAM20, a 1786 bp non-coding DNA fragment was amplified using the primers: F20_non-coding_fwd, 5′-GGAACCGGTTTGATGTCCATTT-3′, and F20_non-coding_rev, 5′-GCTATTGCATGCGCTTAATGA-3′. For *E. coli*, a 1702 bp fragment was amplified using the primers: 5′-AGCCTTATTCTTCATCGTTTTT-3′ and 5′-CATCATTGAGTGCGGCATTTTC-3′. For *L. reuteri*, fragments of 1610 bp (5′-GTTAGGTTGAGGGCCAGGTT-3′ and 5′-GTTGGGTTGGAGGGGTAGTT-3′) and 1791 bp (5′-AATGGACGCTAAAATGGAAGT-3′ and 5′-AGGGGAAAAGAATATGAGAAAT-3′) was amplified. For preparation of eukaryotic DNA, human pharyngeal epithelial FaDu cells were trypsinized, centrifuged at 8000 *g* for 10 min, washed with PBS and resuspended in TE buffer with 0.1% Triton x100 (Sigma-Aldrich). The cell suspension was boiled at 100°C for 5 min and then centrifuged at 13000 *g* for 10 min. The supernatant was collected and stored at −20°C or used immediately for amplification. A 1735 bp fragment was amplified using primers (5′-CAGTTGGTGCGGGAAGGAGT-3′ and 5′-GGAAGAGAAGGAAAGGAGGGCT-3′).

Two hundred ng of purified DNA fragments was incubated with hBD2 (final concentration of 0.1, 1, 2.5, 5, or 10 μM) or BSA (final concentration of 0.1, 1, 5, or 10 μM) in binding buffer (5% glycerol, 10 mM Tris/HCl (pH 8), 1 mM EDTA, 1 mM dithiothreitol, 20 mM KCl, 50 μg/ml BSA). The reaction mixture was incubated at room temperature for 1 h and subjected to 1% agarose gel electrophoresis. Gels were stained with ethidium bromide, and images were captured using a Gel Doc 2000 Imaging System (Bio-Rad).

### Killing Assay in the Presence of DNA

To determine whether the presence of DNA affected the ability of hBD2 to kill *N. meningitidis*, we used a previously described protocol (Jones et al., 2013). hBD2 at a final concentration of 5 μM was preincubated with 0.1, 0.5, or 1 μg of the DNA fragment described above for 15 min at room temperature. Log phase bacteria, prepared as performed in the killing assay above, were added to the hBD2-DNA mixture and incubated for 3 h. Bacteria were serially diluted, plated for viable counts and enumerated the next day.

### DNA Quantification in Culture Supernatants

Log-phase bacteria were prepared as performed in the killing assay above, but without peptide. After 3 h of incubation, samples were centrifuged at 5000 × *g* for 3 min, and the supernatants were collected and incubated with Quant-iT PicoGreen dsDNA dye (Thermo Fisher Scientific) at a 1:1 ratio in black-walled, clear bottom microplates. Fluorescence was measured using a Spectramax i3x microplate reader with an excitation wavelength of 485 nm and an emission wavelength of 535 nm. DNA was quantified based on a Lambda DNA standard.

### Biofilm Assay and eDNA Content Under Static Condition

To measure the stability of meningococcal biofilm upon treatment with hBD2, a static biofilm assay previously described was used with some modifications (Engman et al., 2016; Sigurlásdóttir et al., 2019). Bacteria from plates were suspended to an OD of 0.05 in supplemented GC broth. 100 μl of bacterial suspension was added in quadruplicate to either clear or black-walled polystyrene plates. Plates were incubated for 24 h at 37°C and 5% CO_2_ under static conditions, washed in PBS, and incubated for 1 h or 3 h with 100 μl of either 5 μM BSA, 5 μM hBD2, 200 μg/ml DNase or 0.5% Triton X-100.

For measurement of the total biofilm mass clear plates were washed in PBS, stained with 0.3% crystal violet for 2 min, washed twice with PBS, and solubilized in 30% acetic acid. A crystal violet standard was included with known concentration and the absorbance was measured at 630 nm. The eDNA content was measured in the black-walled plates using Quant-iT PicoGreen dsDNA dye. 100 μl TE buffer and PicoGreen solution (1 μl:199 μl) was added into the biofilm and pipetted 10 times for it to dissolve. eDNA was quantified with a lambda DNA standard and fluorescence was measured at excitation wavelength 485 nm and emission wavelength 535 nm. Measurement of both plates was performed in a Spectramax i3x microplate reader. OD_600_ and viable counts were assessed after the treatments. For viable counts bacterial suspensions were serially diluted, plated and counted the next day. Before plating for viable counts, bacteria were suspended and vortexed to dissolve aggregates, and then examined by microscopy to verify the absence of any aggregates.

### Statistical Analysis

Testing of statistical significance was performed in Graph Pad Prism (version 5). Two-tailed and unpaired Student’s *t*-tests were used to compare differences between two groups. Analysis of variance (ANOVA) with Bonferroni’s *post hoc* test was used to compare differences between multiple groups. *P*-values below 0.05 were considered statistically significant.

## Results

### Human Beta-Defensin 2 Kills Actively Growing *N. meningitidis* Over the Course of Hours

**FIGURE 1:**
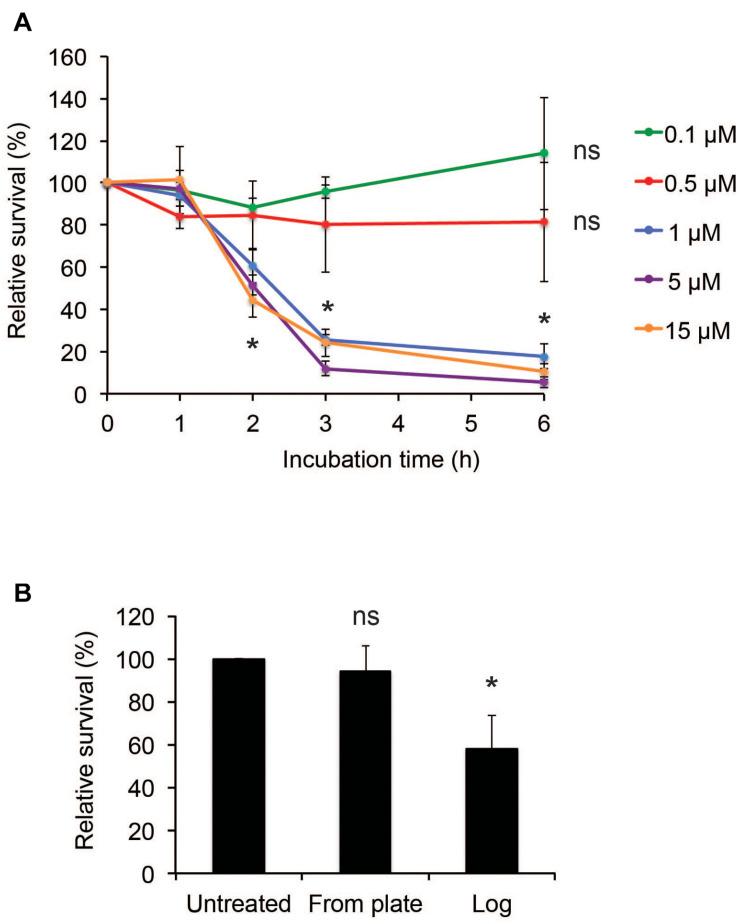
*Neisseria meningitidis* is killed by hBD2. **(A)** Survival of log-phase bacteria incubated with hBD2 at 0.1, 0.5, 1, 5, or 15 μM for 1, 2, 3, or 6 h. **(B)** Survival of bacteria, taken from 18 h over night plates directly or grown to log phase, incubated with 5 μM hBD2 for 2 h. Viable counts were determined by plating. Survival is expressed relative to the untreated control. Data are represented as the mean survival, with error bars representing the standard deviation. Significance was tested against the untreated control. The assay was performed in triplicate three times. *P < 0.05; ns, non-significant.

To assess the susceptibility of *N. meningitidis* to hBD2, killing assays were performed using a range of 0.1 μM to 15 μM hBD2. It has been demonstrated that basal levels of hBD2 in lung fluid are 0.5 μM (Schaller-Bals et al., 2002), and in epidermal tissue 3.5-16 μM (Liu et al., 2002), but in inflammatory conditions, such as in psoriatic skin lesions, the levels of hBD2 can be induced to 25 μM-150 μM (Ong et al., 2002). Log-phase *N. meningitidis* FAM20 was incubated with hBD2 peptide for up to 6 h. We observed a killing effect after 2 h, but not at earlier time points (Figure 1A). At concentrations of 1 μM or higher, bacterial survival was significantly reduced to approximately 50% compared to untreated bacteria, and after 3 h of incubation, the bacterial survival was further reduced to approximately 20% (Figure 1A). Incubation for 6 h did not much increase the killing effect compared to 3 h. The bacterial survival was similar in concentrations from 1 μM hBD2 and higher, supporting that killing by hBD2 is not directly concentration-dependent but rather that hBD2 levels need to reach a threshold level to be bactericidal. Concentrations of 0.1 μM and 0.5 μM did not significantly reduce bacterial survival. To investigate the effect of hBD2 on different growth stages of *N. meningitidis*, we compared bacteria grown to log phase with bacteria that were collected directly from plates and treated with 5 μM hBD2 for 2 h. As shown in Figure 1B, bacteria taken directly from plates were not affected by hBD2, indicating that log phase cells are more susceptible to killing by hBD2 than those from over night plates. We also compared bacteria grown in liquid to stationary phase with log phase and found that bacteria in stationary phase were not susceptible to hBD2 (Supplementary Figure 1). Taken together, the data demonstrate that hBD2 kills log phase *N. meningitidis*.

### Human Beta-Defensin 2 Kills *N. meningitidis* Without Increasing Outer Membrane Permeability

**FIGURE 2:**
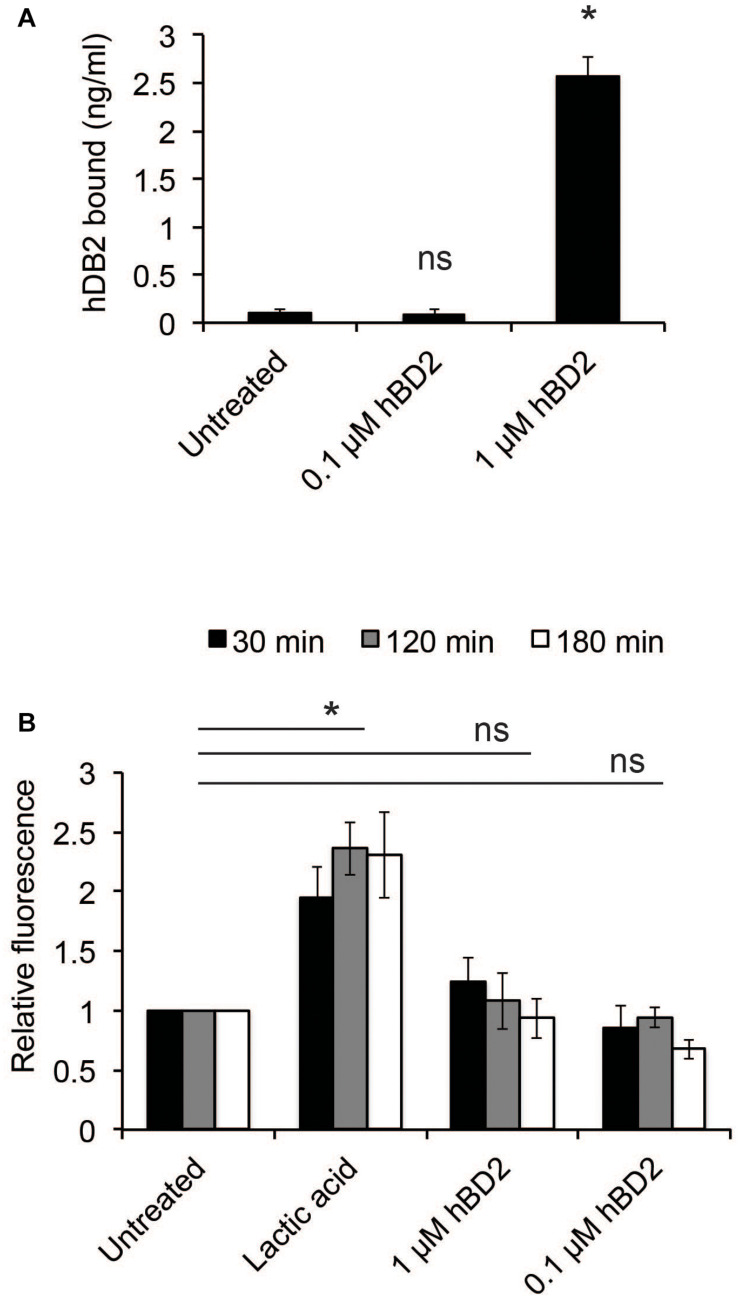
Human beta-defensin 2 binds *N. meningitidis* and induces membrane permeability. **(A)** Log-phase bacteria were incubated with hBD2 (0.1 or 1 μM) for 3 h. Bacteria were washed with PBS, and bound hBD2 was determined by ELISA. The assay was performed in triplicate at least three times. **(B)** Outer-membrane permeabilization was assessed by NPN fluorescence. Log-phase bacteria were incubated with NPN (10 μM) and hBD2 (0.1 μM and 1 μM) for 3 h. Lactic acid was used as a positive control. Background fluorescence (NPN in the absence of bacteria or hBD2) was subtracted. Values are expressed relative to the untreated control. The assay was performed in duplicate at least three times. Data are represented as the mean values, with error bars representing the standard deviation. Significance was tested against the untreated control. *P < 0.05; ns, non-significant.

We next examined whether hBD2 could bind to bacteria. Bacteria were incubated with either 0.1 μM (non-lethal) or 1 μM hBD2 (lethal) for 3 h; then, the level of bound hBD2 was assessed by ELISA. Bacteria incubated with 1 μM hBD2 showed strong and significant hBD2 binding, while treatment with 0.1 μM resulted in a signal similar to the untreated control (Figure 2A). These data show that hBD2 is indeed able to bind to *N. meningitidis*. To examine the effect of hBD2 on bacterial membrane integrity, we measured outer membrane permeability after treatment with 0.1 μM or 1 μM hBD2. Outer membrane permeability was measured using NPN, a hydrophobic probe that fluoresces only in hydrophobic environments. The uptake of NPN into the compromised membrane is therefore a measure of outer membrane permeability (Alakomi et al., 2000). Compared to lactic acid, which is known to permeabilize gram-negative bacteria (Alakomi et al., 2000), we did not detect a significant increase in outer membrane permeability at any of the time points tested (30 min, 2 h or 3 h) (Figure 2B). We further confirmed the reliability of the NPN-assay by showing that the antimicrobial peptide LL-37, known to efficiently kill *N. meningitidis* (Jones et al., 2009), strongly induced membrane permeability (Supplementary Figure 2).

These data indicate that hBD2 may induce bacterial killing by non-membrane disruptive mechanisms.

### Human Beta-Defensin 2 Causes a Shift in Meningococcal DNA Content

**FIGURE 3:**
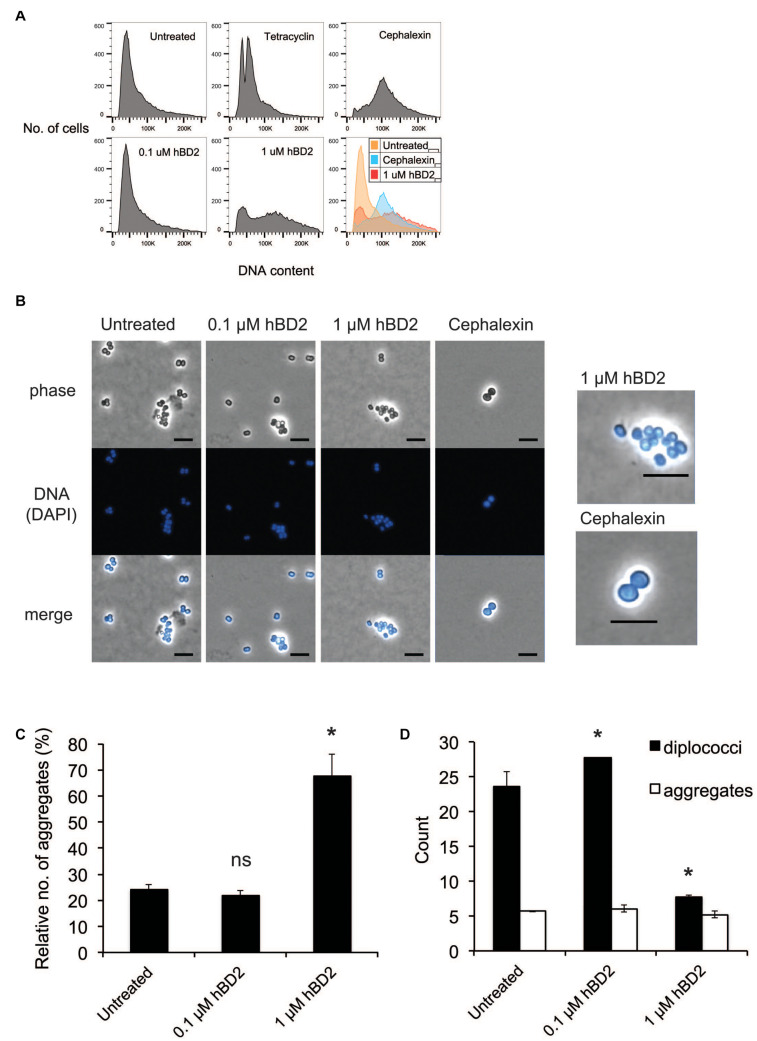
*Neisseria meningitidis* diplococci are more susceptible to hBD2. **(A)** Flow cytometry analysis of *N. meningitidis* in the presence of hBD2. Log-phase bacteria were incubated with hBD2 (0.1 or 1 μM), tetracycline (2 μg/ml), or cephalexin (10 μg/ml) for 2 h. Bacteria were washed in PBS, fixed, permeabilized, and resuspended in sodium citrate buffer. DNA was stained with SYTOX Green, and DNA content was analyzed by flow cytometry. The assay was performed in single samples three times. **(B)** Microscopy of *N. meningitidis*. Log-phase bacteria were incubated with hBD2 (0.1 or 1 μM) or cephalexin (10 μg/ml) for 2 h. Bacteria were washed in PBS once, fixed, permeabilized and resuspended in PBS. DNA was stained with DAPI, and bacteria were visualized by fluorescence microscopy. On the right side are enlarged images of indicated treatments. The scale bar represents 5 μm. Representative images are shown. **(C,D)** Microscopy images were used to enumerate diplococci bacteria and bacteria in aggregates, where aggregates were defined as clusters of 4 or more cells. Counts are expressed as relative numbers of aggregates compared to diplococci **(C)** or as absolute numbers **(D)**. The assay was performed two times with at least 12 images captured per sample. Data are represented as the mean values, with error bars representing the standard deviation. Significance was tested against the untreated control. *P < 0.05; ns, non-significant.

Since hBD2 did not seem to act on outer membrane integrity, we hypothesized that hBD2 targets intracellular components. It has been shown previously that AMPs are capable of affecting DNA synthesis (Gottschalk et al., 2013). To explore potential intracellular targets of hBD2, we next considered the effect of hBD2 on the cell cycle of *N. meningitidis*. We used flow cytometry of SYTOX green-stained bacteria and compared hBD2 treatment to tetracycline and cephalexin treatment. Tetracycline inhibits the initiation of DNA replication but allows the ongoing round of replication to finish, producing fully replicated chromosomes that are visualized as an integer number of peaks, while cephalexin inhibits cell division and will result in cells with increased DNA content (Boye and Lobner-Olesen, 1991; Tobiason and Seifert, 2010). We incubated the cells for 2 h with hBD2 to have at least 50% viable and detectable bacteria in the assay. Treatment with a non-lethal concentration of hBD2 (0.1 μM) resulted in an identical chromosome distribution compared with untreated cultures, whereas treatment with 1 μM hBD2 resulted in a large portion of the population exhibiting an increased amount of DNA per bacteria (Figure 3A). Since the peak shift observed for 1 μM hBD2 resembled that of cephalexin, we hypothesized that hBD2 might interfere with bacterial cell division. Alternatively, hBD2 may promote bacterial aggregation, which would also be detected as particles with increased DNA content. To verify, we used a non-aggregative Δ*pilE* mutant and treated with cephalexin and 1 μM hBD2. We found that Δ*pilE* mutant showed similar pattern with and without hBD2, whereas cephalexin induced a shift in DNA content (Supplementary Figure 3), supporting that aggregation might be an explanation for increased DNA content after hBD2 treatment of the wild-type bacteria.

### Lethal hBD2 Concentrations Reduce Planktonic Bacteria but Leave Aggregates Intact

To assess whether the increased DNA content in the flow cytometry was due to bacterial aggregates, we examined the bacteria by microscopy after treatment with 0.1 μM or 1 μM hBD2 for 2 h. To visualize bacteria, we used phase contrast microscopy and fluorescence microscopy after DAPI staining. We found that the surviving bacteria treated with 1 μM hBD2 were more often in aggregates compared to the control (Figure 3B). Aggregates were defined as 4 or more cells. Visual quantification of planktonic diplococci versus aggregates confirmed that the relative number of aggregates was significantly higher with 1 μM hBD2 compared to the untreated control (Figure 3C). Interestingly, the number of diplococci/planktonic bacteria was significantly reduced for bacteria treated with 1 μM hBD2, although the number of aggregates was similar for all treatments (Figure 3D). Compared to the control, the size of the aggregates remained the same after the 1 μM hBD2 treatment (data not shown). These data indicate that the aggregates detected in the samples treated with 1 μM hBD2 may correspond to the increase in DNA content detected by flow cytometry, rather than an inhibition of cell division (Figure 3A). This is supported by microscopic visualization of cephalexin-treated *N. meningitidis* that showed an increase in cell size compared to 1 μM hBD2 treated bacteria (Figure 3B). However, it is possible that hBD2 affects cellular division in a different way than cephalexin. Although data do not exclude the possibility that hBD2 also affects meningococcal cell division, the results suggest that bacterial aggregates may be better protected against hBD2 than planktonic bacteria.

### Aggregation-Deficient Meningococci Are More Susceptible to hBD2 Compared to Wild-Type Meningococci

**FIGURE 4:**
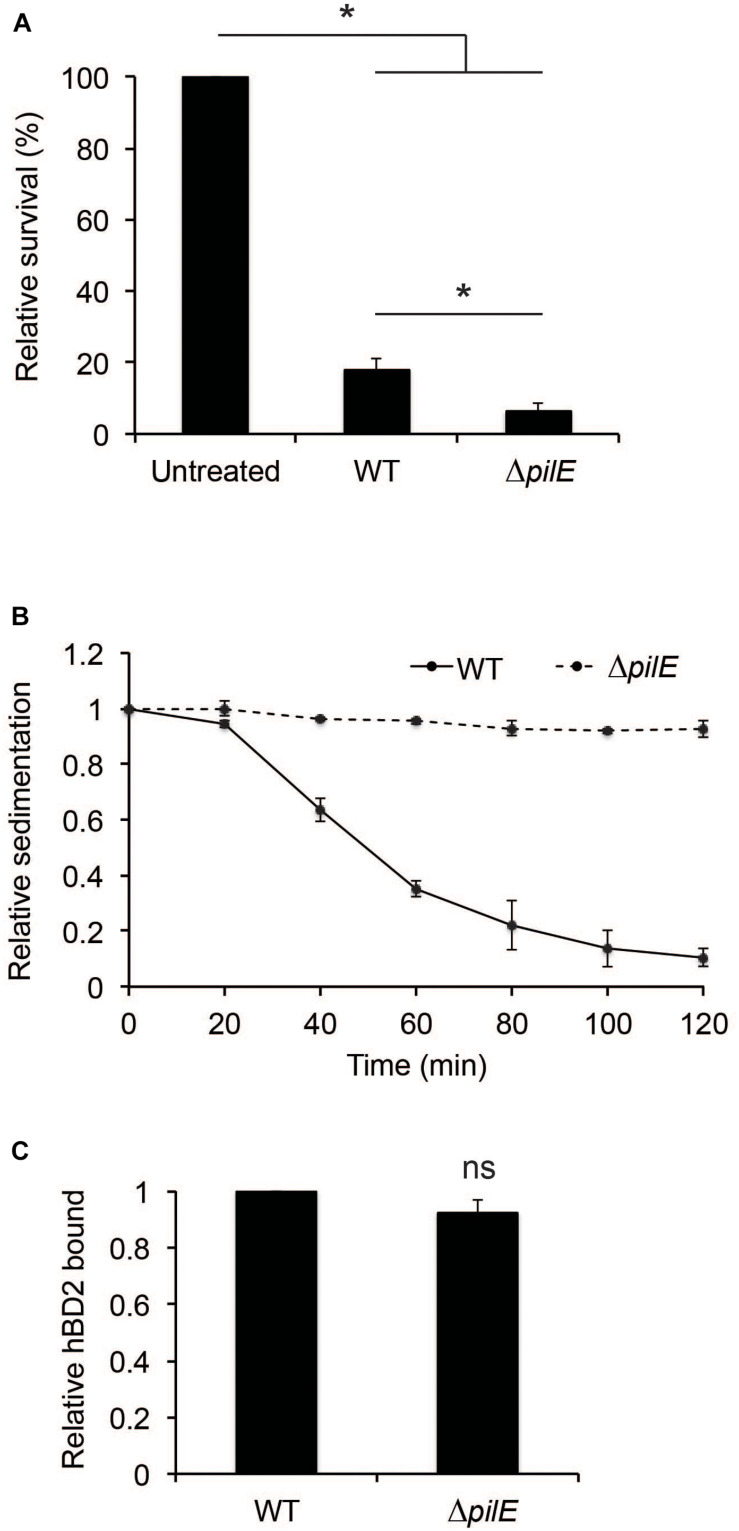
Human beta-defensin 2-binding and aggregation of wild-type and Δ*pilE* bacteria. **(A)** Survival of log-phase bacteria of wild-type (WT) and Δ*pilE* incubated with 1 μM hBD2 for 3 h. Viable counts were determined by plating. Values are expressed as the mean survival relative to the untreated control. The assay was performed in triplicate at least three times. **(B)** Aggregation of bacteria determined by a sedimentation assay. WT and the Δ*pilE* mutant were grown to log phase, pelleted, resuspended in modified DMEM assay medium, and incubated under static conditions at room temperature. Optical density measurements of the top layer were made at 20 min intervals. Values are expressed relative to the 0 min timepoint. The assay was performed in single samples at least three times. **(C)** WT and Δ*pilE* were grown to log phase and then incubated with 1 μM hBD2 for 3 h. Bacteria were washed with PBS once, and bound hBD2 was determined by ELISA. Values are expressed relative to WT. Data are represented as the mean values, with error bars representing the standard deviation. The assay was performed in triplicate at least three times. Significance was tested against the untreated control, or against WT, as indicated. *P < 0.05; ns, non-significant.

To further investigate the contribution of bacterial aggregation to protect against hBD2-mediated killing, we included a Δ*pilE* mutant. The Δ*pilE* mutant does not express type IV pili and is therefore unable to aggregate. We grew the wild-type strain FAM20 and the isogenic Δ*pilE* mutant to log phase and incubated these strains with 1 μM hBD2 for 3 h. The non-piliated mutant was three-fold more sensitive to hBD2 compared to wild-type bacteria (Figure 4A). We also tested a hyper-aggregative *pilT* deletion mutant, which showed to be more resistant to hBD2 killing (Supplementary Figure 4), supporting that aggregated bacteria survive hBD2 better. To confirm the lack of aggregation in the experimental conditions used for the killing assay, we performed a sedimentation assay. Indeed, Δ*pilE* bacteria did not sediment (Figure 4B), confirming the non-aggregative phenotype. In addition, we did not observe any difference in hBD2 binding between the Δ*pilE* mutant and WT in ELISA assays (Figure 4C), indicating that hBD2 binding in the presence or absence of pili is not correlated with the killing effect. Taken together, these data suggest that the non-aggregative Δ*pilE* mutant is more susceptible to hBD2 killing, supporting that hBD2 is more lethal to non-aggregated bacteria.

### Human Beta-Defensin 2 Binds Meningococcal DNA in a Dose-Dependent Manner

**FIGURE 5:**
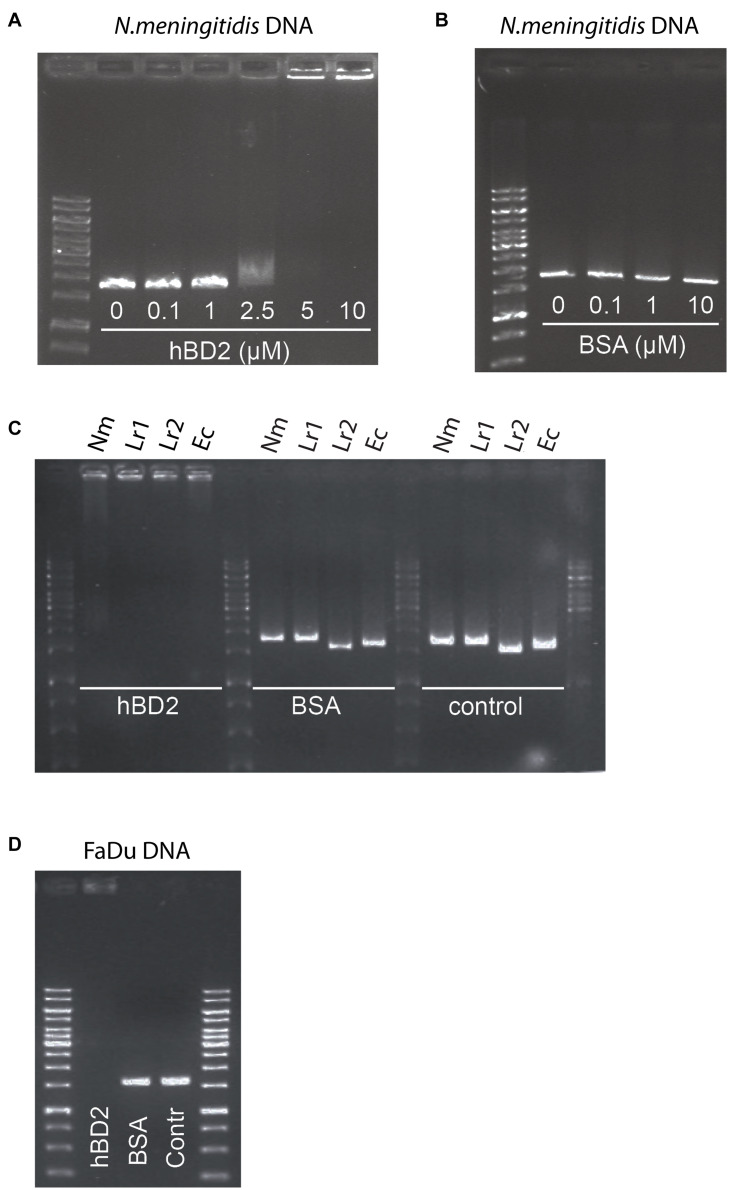
Human beta-defensin 2 binds both bacterial and human DNA *in vitro*. To assess the *in vitro* binding of hBD2 to DNA, an electrophoretic mobility shift assay was performed. **(A)** *N. meningitidis* DNA, as a 1786 bp non-coding DNA fragment, was incubated with hBD2 (0.1, 1, 2.5, 5 or 10 μM) for 1 h. **(B)** BSA (0.1, 1 or 10 μM) was used as a negative control. **(C)** *N. meningitidis* DNA (Nm) as a fragment of 1786 bp, *L. reuteri* DNA as two fragments of 1610 bp (Lr1) and 1791 bp (Lr2) and an *E. coli* DNA fragment (Ec) of 1702 bp, were incubated with hBD2 at a concentration of 5 μM for 1 h. BSA at a concentration of 5 μM was used as a negative control and incubated with the same DNA fragments for 1 h. Bacterial DNA fragments without peptide served as second negative control (control). **(D)** Human pharyngeal epithelial cell FaDu DNA as a fragment of 1735 bp was incubated with hBD2 at a final concentration of 5 μM for 1 h. Incubation of FaDu DNA fragment with BSA at a final concentration 5 μM and without peptide (Contr) served as negative control. After incubation, samples were run on an agarose gel. Representative images are shown. The assays were performed twice.

Bacteria have evolved numerous mechanisms to evade AMPs (Cole and Nizet, 2016). It has been shown that extracellular DNA of *Haemophilus influenzae* can bind to hBD3 and thus neutralize the antibacterial activity of the peptide (Jones et al., 2013). Since hBD2 and hBD3 share a number of structural features, we hypothesized that hBD2 may behave in a similar manner. We therefore assessed whether hBD2 is able to interact with meningococcal DNA using an electrophoretic mobility shift assay with increasing concentrations of hBD2. DNA migration through the gel was reduced in a dose-dependent manner. At 2.5 μM, hBD2-DNA was partially able to migrate through the gel, while at 5 μM, hBD2-DNA was no longer able to migrate into the gel and was stuck in the well (Figure 5A), suggesting formation of larger hBD2-DNA complexes. The mobility of DNA was not altered when the DNA was incubated with BSA (Figure 5B), which served as a negative control. Next, we evaluated whether hBD2 could bind to DNA from *L. reuteri* and *E. coli* by electrophoretic mobility shift assays as above. We found that hBD2 interacted with DNA, not only from meningococci, but also from the other bacterial species (Figure 5C). Further, an additional experiment using DNA from human epithelial cells demonstrated that hBD2 peptide also can bind DNA from human cells (Figure 5D). Together, these results indicate that hBD2 is able to bind DNA from both bacterial and human cells.

### Meningococcal DNA Inhibits hBD2 Antibacterial Activity Against *N. meningitidis*

**FIGURE 6:**
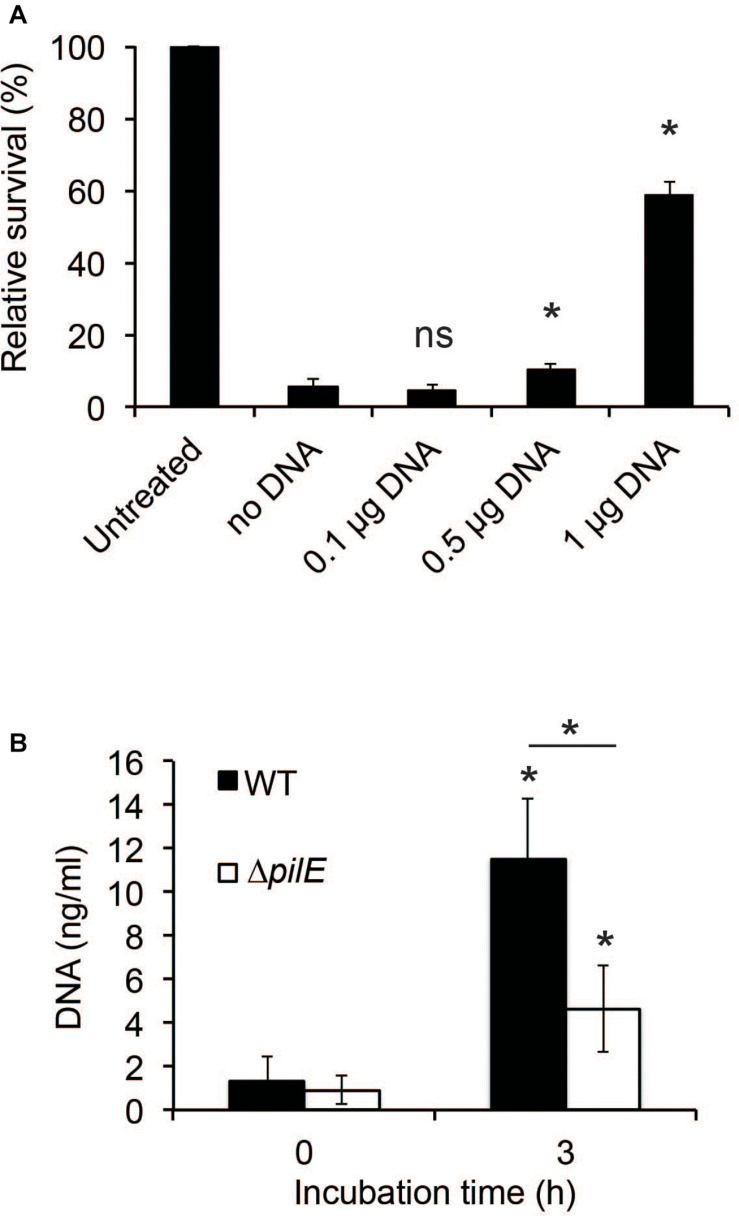
The presence of DNA reduces hBD2 antibacterial activity. **(A)** *N. meningitidis* DNA (0.1, 0.5, or 1 μg) was incubated with 5 μM hBD2 for 15 min, subsequently added to log-phase bacteria and incubated for 3 h. Viable counts were determined by plating. Values are expressed as the mean survival relative to the untreated control. Significance was tested against the sample without DNA. The assay was performed in triplicate at least three times. **(B)** Log-phase WT and Δ*pilE* cells were incubated untreated for 3 h. Detection of eDNA in the supernatants was determined by a fluorescence-based Quant-iT PicoGreen DNA assay kit. Significance was tested against the 0 h time-point and as indicated. The assay was performed in triplicate at least three times. Data are represented as the mean values, with error bars representing the standard deviation. *P < 0.05; ns, non-significant.

Since interactions between bacteria and AMPs rely on charge (Brogden, 2005), the binding of hBD2 to DNA will likely have consequences for its antibacterial activity, as has also been shown previously for hBD3 against *Haemophilus influenzae* (Jones et al., 2013). We preincubated hBD2 with increasing concentrations of meningococcal DNA before adding the hBD2-DNA mixture to log-phase bacteria to quantify bacterial killing. Since we showed complete hBD2-DNA binding at 5 μM hBD2, we performed the killing assay at this concentration. We observed that the killing activity of hBD2 was significantly reduced in the presence of DNA in a dose-dependent manner, starting at 0.5 μg DNA, compared to bacteria that had been treated with hBD2 only (Figure 6A). Thus, meningococcal DNA inhibits the antibacterial effect of hBD2.

To determine if DNA was released by *N. meningitidis* during growth, we grew bacteria for 3 h and found that the amount of extracellular DNA (eDNA) increased during incubation (Figure 6B). To determine if the amount of eDNA released by the Δ*pilE* mutant might contribute to its increased sensitivity, we also assessed the eDNA released from the Δ*pilE* mutant and found that the mutant released less than the wild-type (Figure 6B). Based on these results, we speculate that eDNA release by *N. meningitidis* and its subsequent binding to hBD2 could represent a bacterial evasion mechanism against hBD2-mediated killing.

### Meningococcal Biofilms Are Affected by hBD2

**FIGURE 7:**
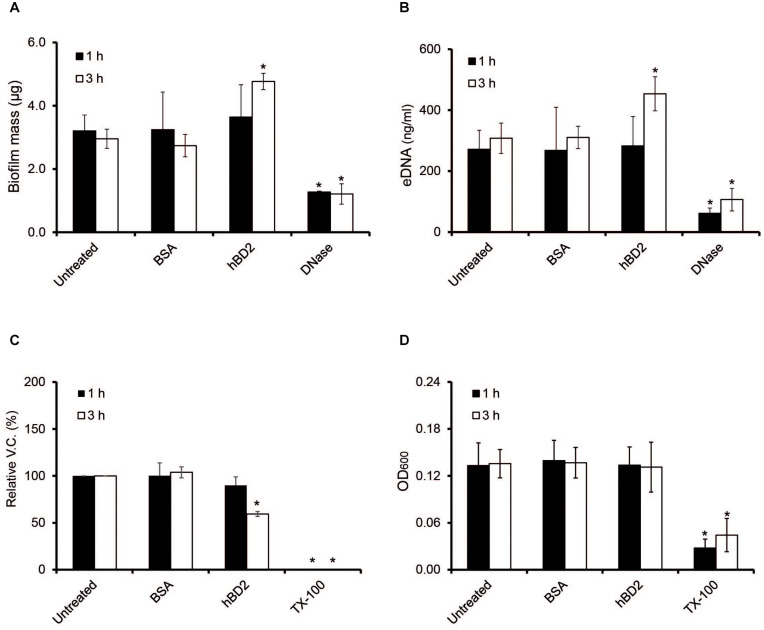
Meningococcal biofilms after hBD2 treatment. Meningococcal biofilms were allowed to form under static conditions for 24 h before incubation with 5 μM BSA or 5 μM hBD2. **(A)** Biofilm mass was detected by crystal violet. **(B)** Detection of eDNA in meningococcal biofilm was performed using Quant-iT PicoGreen DNA assay kit. **(C)** Biofilm was dissolved and viable bacteria quantified by plating. **(D)** OD_600_ of dissolved biofilm. Treatment with 200 μg/ml DNase I was used as negative control in panels **(A,B)**, and 0.5% Triton X-100 (Tx-100) was used as negative control in panels **(C,D)**. DNase I was only used as negative control for the measurement and was never added together with hBD2. Data are represented as the mean values, with error bars representing the standard deviation. All experiments were performed in quadruplicates at least three times. Significance was compared with the untreated biofilm at 1 or 3 h. *P < 0.05.

Biofilms are known to protect bacteria against environmental stressors, and a major component of the meningococcal biofilm is eDNA (Lappann et al., 2010). Since certain AMPs, for example LL-37, have been shown to have antibiofilm effects (Overhage et al., 2008), we next assessed whether hBD2 might affect meningococcal biofilms. We added 5 μM hBD2 to the biofilm and measured total biofilm mass, eDNA content and bacterial viable count. The biofilm mass and eDNA level remained unaffected at 1 h, however, at 3 h post-incubation both biofilm mass and eDNA content exhibited a slight but significant increase (Figures 7A,B). Indeed, treatment with 5 μM hBD2 led to a significant decrease (60%) of viable bacteria at 3 h (Figure 7C). The OD remained constant at both time points, supporting the non-membrane disruptive killing mechanism (Figure 7D). In summary, data indicate that hBD2 does not have an antibiofilm effect on the biofilm mass but can significantly reduce the number of live bacteria in a biofilm. However, as an increase of eDNA was shown, this accumulation could protect surviving bacteria, supporting the hypothesis that meningococcal eDNA functions as an evasion mechanism against hBD2 killing. Thus, the data suggest that bacteria in biofilm are killed by hBD2 and the remaining cells could be protected with the subsequent eDNA increase.

## Discussion

In this study, we examined the antibacterial effect of hBD2 on one strain of *N. meningitidis*. We show that hBD2 binds to and kills actively growing *N. meningitidis*. The lethal effect was not immediate but emerged after more than 1 h of incubation with the peptide. NPN assays did not detect any significant changes in outer membrane integrity during hBD2 treatment, supporting a lengthier type of killing mechanism. Incubation with lethal doses of hBD2 decreased the presence of diplococci but kept the number of bacterial microcolonies/aggregates constant, indicating that meningococcal aggregates were better protected against hBD2 killing. Furthermore, we provide evidence that *N. meningitidis* DNA binds hBD2 and that meningococcal DNA inhibits the lethal effect of hBD2 in a dose-dependent manner. In summary, the data indicate that aggregation and the presence of extracellular DNA are possible evasion mechanisms against hBD2-mediated killing.

Antimicrobial peptide-mediated killing of bacteria can occur by membrane disruptive or non-membrane disruptive mechanisms (Brogden, 2005). We did not detect permeability of the outer membrane during treatment with 1 μM hBD2, indicating that the bacterial killing by hBD2 does not alter membrane integrity at the tested time points. While membrane disruptive killing usually occurs in the range of several minutes, non-membrane disruptive killing can take up to a few hours (Jacob et al., 2014). The observation that hBD2 must be incubated for more than an hour before inducing *N. meningitidis* killing therefore further supports a model where hBD2 targets intracellular components.

Many studies have shown that bacterial aggregation, including biofilm formation, protects bacteria against antimicrobials, primarily due to limited antimicrobial penetration, as well as a lowered metabolic rate (Secor et al., 2018; Yasir et al., 2018). Using microscopic analysis of *N. meningitidis* exposed to hBD2, we found that bacteria in aggregates were better protected against hBD2-mediated killing. Indeed, we observed that non-aggregating pilus-deficient bacteria were more susceptible to hBD2. It is important to note that the sedimentation assay carried out to verify aggregation phenotype was performed in the absence of hBD2, and it is possible that bacteria aggregate differently during exposure to hBD2.

In this work, we found that hBD2 binds *N. meningitidis* DNA *in vitro*. In control experiments, hBD2 did not bind BSA. Further, hBD2 also interacted with DNA from both *E. coli* and *L. reuteri*, as well as DNA from human cells. This indicates that the hBD2-DNA interaction is non-specific and is based on charge, as suggested previously (Gottschalk et al., 2013; Phan et al., 2015). This implies that hBD2 may target not only DNA but also any negatively charged molecule it encounters in the cytoplasm, such as mRNA or proteins. Indeed, AMP-mediated bacterial killing by inhibiting ribosome activity, protein translation, and enzymatic activity are known to occur (Otvos et al., 2000; Gagnon et al., 2016), although these effects are not necessarily due to charge interactions. It was recently shown that hBD2 enters the cytoplasm of *E. coli* and exhibits a killing effect in 2 h (Mathew and Nagaraj, 2017). It remains to be determined whether hBD2 has intracellular targets in meningococci.

We showed that the binding of hBD2 to meningococcal DNA reduces hBD2 antibacterial activity. Since eDNA release by *N. meningitidis* can occur through autolysis (Lappann and Vogel, 2010), it might be that *N. meningitidis* autolysis represents an evasion strategy against the effects of hBD2. In *N. gonorrhoeae* the Type IV secretion system is known to secrete chromosomal DNA (Hamilton et al., 2005), and Type IV secretion systems have been found in *N. meningitidis* (Snyder et al., 2005; Woodhams et al., 2012; Pachulec et al., 2014; Calder et al., 2020) and *H. influenzae* (Jurcisek et al., 2017). Therefore, it might be that both type IV secretion systems and autolysis may aid in bacterial survival in the presence of hBD2. It is possible that the release of DNA from the bacteria explains the stagnation in killing between 3 h and 6 h of incubation with hBD2 (see Figure 1A). Incubation of meningococcal biofilms with hBD2 for 3 h only reduced viable bacteria to 60% (Figure 7), compared to 10% for bacteria not in biofilms (Figure 1), suggesting that eDNA in biofilms may protect against hBD2 killing. During infection external stimuli may trigger hBD2 and we have previously shown that *Lactobacillus* strains trigger hBD2 expression in epithelial cells (Wassing et al., 2021). The data suggest that introduction of hBD2 to biofilms may not affect the biofilm mass, but it may kill active bacteria, which could release eDNA that might result in protection of the remaining bacteria from hBD2. However, other multiple negatively charged molecules present in the biofilm may also be important in binding up hBD2. It remains to determine if hBD2, in addition to eDNA, also binds to for example capsule, outer membrane vesicles or peptidoglycan. In the future, it would be interesting to evaluate whether hBD2-treated meningococci exhibit increased autolysis compared to untreated bacteria. To explore whether *N. meningitidis* responds to the presence of hBD2 by inducing autolysis, expression profiles of autolysin genes, such as the lytic transglycosylases (MltA and MltB) (Lappann and Vogel, 2010), could be assessed in experiments of hBD2 exposure.

It is known that beta-defensins are affected by physiological concentrations of salt and serum (Bals et al., 1998; Harder et al., 2001; Vylkova et al., 2007). For this reason, the *in vivo* relevance of hBD2 has been challenged. It is noteworthy that the local environment can greatly alter AMP activity by influencing peptide conformation and secondary structure (Brogden, 2005). For example, the closely related peptide hBD1 shows markedly increased activity when in a reduced state compared to an oxidized state (Schroeder et al., 2011). Furthermore, inactivation by salt can be overcome by increasing AMP concentration (Singh et al., 1998), which can locally reach up to 150 μM for hBD2 during disease state (Ong et al., 2002). It is possible that synthetic hBD2 might have a different conformation than the native form, however, since the molecule is short and showed a bactericidal effect, the molecule is most likely in an active conformation.

In summary, hBD2 binds to and kills actively growing *N. meningitidis*. The lethal effect was not immediate, as is known for membrane-disruptive AMPs, but appeared at 2 h postincubation. Bacteria in aggregates survive treatment with lethal concentrations of hBD2 more often. Finally, the binding of hBD2 to extracellular DNA due to bacterial lysis is a possible evasion mechanism against hBD2. In this study, we used a *N. meningitidis* serogroup C strain, in the future it would be interesting to assess whether other serogroups behave in a similar way. With the emerging rise in antibiotic resistance, AMPs have increasingly become the focus as a novel antimicrobial treatment strategy. Although antibiotic resistance to *N. meningitidis* does not yet pose a problem, the widespread antimicrobial resistance exhibited by *N. gonorrhoeae*, a closely related strain, may increase the likelihood of the emergence of meningococcal resistant clones (Dillon et al., 1983). Here, we highlight the complexity of bacteria-AMP interactions, as well as possible bacterial evasion strategies against defensins. Our findings provide new directions in which to focus future research on the development of AMP-derived therapeutic applications.

## Data Availability Statement

The raw data supporting the conclusions of this article will be made available by the authors, without undue reservation.

## Author Contributions

GW, KL, SS, JF, KS, A-CL, KJ, and A-BJ conceived and designed the experiments. GW, KL, SS, JF, KS, and NI performed the experiments. GW, KL, SS, JF, A-CL, KJ, and A-BJ analyzed the data and wrote the manuscript. All authors contributed to the article and approved the submitted version.

## Conflict of Interest

The authors declare that the research was conducted in the absence of any commercial or financial relationships that could be construed as a potential conflict of interest.

## References

[B1] Alakomi H. L. Skytta E. Saarela M. Mattila-Sandholm T. Latva-Kala K. Helander I. M. (2000). Lactic acid permeabilizes gram-negative bacteria by disrupting the outer membrane. *Appl. Environ. Microbiol.* 66 2001–2005. 10.1128/aem.66.5.2001-2005.2000 10788373 PMC101446

[B2] Ali R. S. Falconer A. Ikram M. Bissett C. E. Cerio R. Quinn A. G. (2001). Expression of the peptide antibiotics human beta defensin-1 and human beta defensin-2 in normal human skin. *J. Invest. Dermatol.* 117 106–111. 10.1046/j.0022-202x.2001.01401.x 11442756

[B3] Arenas J. Tommassen J. (2017). Meningococcal biofilm formation: let’s stick together. *Trends Microbiol.* 25 113–124. 10.1016/j.tim.2016.09.005 27712951

[B4] Bals R. Wang X. Wu Z. Freeman T. Bafna V. Zasloff M. (1998). Human beta-defensin 2 is a salt-sensitive peptide antibiotic expressed in human lung. *J. Clin. Invest.* 102 874–880. 10.1172/jci2410 9727055 PMC508952

[B5] Boye E. Lobner-Olesen A. (1991). Bacterial growth control studied by flow cytometry. *Res. Microbiol.* 142 131–135. 10.1016/0923-2508(91)90020-b 1925010

[B6] Brogden K. A. (2005). Antimicrobial peptides: pore formers or metabolic inhibitors in bacteria? *Nat. Rev. Microbiol.* 3 238–250. 10.1038/nrmicro1098 15703760

[B7] Calder A. Menkiti C. J. Cagdas A. Lisboa Santos J. Streich R. Wong A. (2020). Virulence genes and previously unexplored gene clusters in four commensal *Neisseria* spp. isolated from the human throat expand the *Neisserial* gene repertoire. *Microb. Genom.* 6:mgen000423. 10.1099/mgen.0.000423 PMC7643975 32845827

[B8] Chadebech P. Goidin D. Jacquet C. Viac J. Schmitt D. Staquet M. J. (2003). Use of human reconstructed epidermis to analyze the regulation of beta-defensin hBD-1, hBD-2, and hBD-3 expression in response to LPS. *Cell Biol. Toxicol.* 19 313–324. 10.1023/b:cbto.0000004975.36521.c8 14703118

[B9] Christensen H. May M. Bowen L. Hickman M. Trotter C. L. (2010). Meningococcal carriage by age: a systematic review and meta-analysis. *Lancet Infect. Dis.* 10 853–861. 10.1016/s1473-3099(10)70251-6 21075057

[B10] Cole J. N. Nizet V. (2016). Bacterial evasion of host antimicrobial peptide defenses. *Microbiol. Spectr.* 4:10.1128/microbiolsec.VMBF-0006-2015. 10.1128/microbiolspec.VMBF-0006-2015 PMC4804471 26999396

[B11] de Klerk N. Maudsdotter L. Gebreegziabher H. Saroj S. D. Eriksson B. Eriksson O. S. (2016). *Lactobacilli* reduce *Helicobacter pylori* attachment to host gastric epithelial cells by inhibiting adhesion gene expression. *Infect. Immun.* 84 1526–1535. 10.1128/iai.00163-16 26930708 PMC4862695

[B12] Dillon J. R. Pauze M. Yeung K. H. (1983). Spread of penicillinase-producing and transfer plasmids from the gonococcus to *Neisseria meningitidis*. *Lancet* 1 779–781. 10.1016/s0140-6736(83)91846-9 6132128

[B13] Engman J. Negrea A. Sigurlasdottir S. Georg M. Eriksson J. Eriksson O. S. (2016). *Neisseria meningitidis* polynucleotide phosphorylase affects aggregation, adhesion and virulence. *Infect Immun.* 84 1501–1513. 10.1128/iai.01463-15 26930706 PMC4862713

[B14] Gagnon M. G. Roy R. N. Lomakin I. B. Florin T. Mankin A. S. Steitz T. A. (2016). Structures of proline-rich peptides bound to the ribosome reveal a common mechanism of protein synthesis inhibition. *Nucleic Acids Res.* 44 2439–2450. 10.1093/nar/gkw018 26809677 PMC4797290

[B15] Gottschalk S. Ifrah D. Lerche S. Gottlieb C. T. Cohn M. T. Hiasa H. (2013). The antimicrobial lysine-peptoid hybrid LP5 inhibits DNA replication and induces the SOS response in *Staphylococcus aureus*. *BMC Microbiol.* 13:192. 10.1186/1471-2180-13-192 23945181 PMC3751284

[B16] Hamilton H. L. Dominguez N. M. Schwartz K. J. Hackett K. T. Dillard J. P. (2005). *Neisseria gonorrhoeae* secretes chromosomal DNA via a novel type IV secretion system. *Mol. Microbiol.* 55 1704–1721. 10.1111/j.1365-2958.2005.04521.x 15752195

[B17] Harder J. Bartels J. Christophers E. Schroder J. M. (2001). Isolation and characterization of human beta -defensin-3, a novel human inducible peptide antibiotic. *J. Biol. Chem.* 276 5707–5713. 10.1074/jbc.m008557200 11085990

[B18] Helaine S. Carbonnelle E. Prouvensier L. Beretti J. L. Nassif X. Pelicic V. (2005). PilX, a pilus-associated protein essential for bacterial aggregation, is a key to pilus-facilitated attachment of *Neisseria meningitidis* to human cells. *Mol. Microbiol.* 55 65–77. 10.1111/j.1365-2958.2004.04372.x 15612917

[B19] Hoover D. M. Rajashankar K. R. Blumenthal R. Puri A. Oppenheim J. J. Chertov O. (2000). The structure of human beta-defensin-2 shows evidence of higher order oligomerization. *J. Biol. Chem.* 275 32911–32918. 10.1074/jbc.m006098200 10906336

[B20] Jacob B. Kim Y. Hyun J. K. Park I. S. Bang J. K. Shin S. Y. (2014). Bacterial killing mechanism of sheep myeloid antimicrobial peptide-18 (SMAP-18) and its Trp-substituted analog with improved cell selectivity and reduced mammalian cell toxicity. *Amino Acids* 46 187–198. 10.1007/s00726-013-1616-8 24221355

[B21] Joly S. Maze C. Mccray P. B. Jr. Guthmiller J. M. (2004). Human beta-defensins 2 and 3 demonstrate strain-selective activity against oral microorganisms. *J. Clin. Microbiol.* 42 1024–1029. 10.1128/jcm.42.3.1024-1029.2004 15004048 PMC356847

[B22] Jones A. Georg M. Maudsdotter L. Jonsson A. B. (2009). Endotoxin, capsule, and bacterial attachment contribute to *Neisseria meningitidis* resistance to the human antimicrobial peptide LL-37. *J. Bacteriol.* 191 3861–3868. 10.1128/jb.01313-08 19376861 PMC2698406

[B23] Jones E. A. Mcgillivary G. Bakaletz L. O. (2013). Extracellular DNA within a nontypeable *Haemophilus influenzae*-induced biofilm binds human beta defensin-3 and reduces its antimicrobial activity. *J. Innate Immun.* 5 24–38. 10.1159/000339961 22922323 PMC3640559

[B24] Jurcisek J. A. Brockman K. L. Novotny L. A. Goodman S. D. Bakaletz L. O. (2017). Nontypeable *Haemophilus influenzae* releases DNA and DNABII proteins via a T4SS-like complex and ComE of the type IV pilus machinery. *Proc. Natl. Acad. Sci. U.S.A.* 114 E6632–E6641. 28696280 10.1073/pnas.1705508114 PMC5559034

[B25] Kellogg D. S. Jr. Cohen I. R. Norins L. C. Schroeter A. L. Reising G. (1968). *Neisseria gonorrhoeae*. II. Colonial variation and pathogenicity during 35 months in vitro. *J. Bacteriol.* 96 596–605. 10.1128/jb.96.3.596-605.1968 4979098 PMC252347

[B26] Lappann M. Claus H. Van Alen T. Harmsen M. Elias J. Molin S. (2010). A dual role of extracellular DNA during biofilm formation of *Neisseria meningitidis*. *Mol. Microbiol.* 75 1355–1371. 10.1111/j.1365-2958.2010.07054.x 20180907

[B27] Lappann M. Vogel U. (2010). Biofilm formation by the human pathogen *Neisseria meningitidis*. *Med. Microbiol. Immunol.* 199 173–183. 10.1007/s00430-010-0149-y 20376486

[B28] Liu A. Y. Destoumieux D. Wong A. V. Park C. H. Valore E. V. Liu L. (2002). Human beta-defensin-2 production in keratinocytes is regulated by interleukin-1, bacteria, and the state of differentiation. *J. Invest. Dermatol.* 118 275–281. 10.1046/j.0022-202x.2001.01651.x 11841544

[B29] Mathew B. Nagaraj R. (2017). Variations in the interaction of human defensins with *Escherichia coli*: possible implications in bacterial killing. *PLoS One* 12:e0175858. 10.1371/journal.pone.0175858 28423004 PMC5397029

[B30] Mattiuzzo M. Bandiera A. Gennaro R. Benincasa M. Pacor S. Antcheva N. (2007). Role of the *Escherichia coli* SbmA in the antimicrobial activity of proline-rich peptides. *Mol. Microbiol.* 66 151–163. 10.1111/j.1365-2958.2007.05903.x 17725560

[B31] Morand P. (2004). Type IV pilus retraction in pathogenic *Neisseria* is regulated by the PilC proteins. *EMBO J.* 23 2009–2017. 10.1038/sj.emboj.7600200 15103324 PMC404320

[B32] O’Neil D. A. Porter E. M. Elewaut D. Anderson G. M. Eckmann L. Ganz T. (1999). Expression and regulation of the human beta-defensins hBD-1 and hBD-2 in intestinal epithelium. *J. Immunol.* 163 6718–6724. 10586069

[B33] Ong P. Y. Ohtake T. Brandt C. Strickland I. Boguniewicz M. Ganz T. (2002). Endogenous antimicrobial peptides and skin infections in atopic dermatitis. *N. Engl. J. Med.* 347 1151–1160. 10.1056/nejmoa021481 12374875

[B34] Otvos L. Jr. Insug O. Rogers M. E. Consolvo P. J. Condie B. A. Lovas S. (2000). Interaction between heat shock proteins and antimicrobial peptides. *Biochemistry* 39 14150–14159. 10.1021/bi0012843 11087363

[B35] Overhage J. Campisano A. Bains M. Torfs E. C. Rehm B. H. Hancock R. E. (2008). Human host defense peptide LL-37 prevents bacterial biofilm formation. *Infect. Immun.* 76 4176–4182. 10.1128/iai.00318-08 18591225 PMC2519444

[B36] Pace D. Pollard A. J. (2012). Meningococcal disease: clinical presentation and sequelae. *Vaccine* 30(Suppl. 2), B3–B9. 22607896 10.1016/j.vaccine.2011.12.062

[B37] Pachulec E. Siewering K. Bender T. Heller E. M. Salgado-Pabon W. Schmoller S. K. (2014). Functional analysis of the Gonococcal genetic Island of *Neisseria gonorrhoeae*. *PLoS One* 9:e109613. 10.1371/journal.pone.0109613 25340397 PMC4207684

[B38] Phan H. T. Bartelt-Hunt S. Rodenhausen K. B. Schubert M. Bartz J. C. (2015). Investigation of Bovine Serum Albumin (BSA) attachment onto Self-Assembled Monolayers (SAMs) using combinatorial quartz crystal microbalance with dissipation (QCM-D) and Spectroscopic ellipsometry (SE). *PLoS One* 10:e0141282. 10.1371/journal.pone.0141282 26505481 PMC4624694

[B39] Pujol C. Eugene E. Marceau M. Nassif X. (1999). The meningococcal PilT protein is required for induction of intimate attachment to epithelial cells following pilus-mediated adhesion. *Proc. Natl. Acad. Sci. U.S.A.* 96 4017–4022. 10.1073/pnas.96.7.4017 10097155 PMC22412

[B40] Rahman M. Kallstrom H. Normark S. Jonsson A. B. (1997). PilC of pathogenic *Neisseria* is associated with the bacterial cell surface. *Mol. Microbiol.* 25 11–25. 10.1046/j.1365-2958.1997.4601823.x 11902714

[B41] Schaller-Bals S. Schulze A. Bals R. (2002). Increased levels of antimicrobial peptides in tracheal aspirates of newborn infants during infection. *Am. J. Respir. Crit. Care Med.* 165 992–995. 10.1164/ajrccm.165.7.200110-020 11934727

[B42] Schroeder B. O. Wu Z. Nuding S. Groscurth S. Marcinowski M. Beisner J. (2011). Reduction of disulphide bonds unmasks potent antimicrobial activity of human beta-defensin 1. *Nature* 469 419–423. 10.1038/nature09674 21248850

[B43] Secor P. R. Michaels L. A. Ratjen A. Jennings L. K. Singh P. K. (2018). Entropically driven aggregation of bacteria by host polymers promotes antibiotic tolerance in *Pseudomonas aeruginosa*. *Proc. Natl. Acad. Sci. U.S.A.* 115 10780–10785. 10.1073/pnas.1806005115 30275316 PMC6196481

[B44] Selsted M. E. Tang Y. Q. Morris W. L. Mcguire P. A. Novotny M. J. Smith W. (1993). Purification, primary structures, and antibacterial activities of beta-defensins, a new family of antimicrobial peptides from bovine neutrophils. *J. Biol. Chem.* 268 6641–6648. 10.1016/s0021-9258(18)53298-1 8454635

[B45] Sigurlásdóttir S. Wassing G. M. Zuo F. Arts M. Jonsson A. B. (2019). Deletion of D-Lactate Dehydrogenase A in *Neisseria meningitidis* promotes biofilm formation through increased autolysis and extracellular DNA release. *Front. Microbiol.* 10:422. 10.3389/fmicb.2019.00422 30891026 PMC6411758

[B46] Singh P. K. Jia H. P. Wiles K. Hesselberth J. Liu L. Conway B. A. (1998). Production of beta-defensins by human airway epithelia. *Proc. Natl. Acad. Sci. U.S.A.* 95 14961–14966. 10.1073/pnas.95.25.14961 9843998 PMC24558

[B47] Snyder L. A. S. Jarvis S. A. Saunders N. J. (2005). Complete and variant forms of the ‘gonococcal genetic island’ in *Neisseria meningitidis*. *Microbiology* 151 4005–4013. 10.1099/mic.0.27925-0 16339945

[B48] Spinosa M. R. Progida C. Tala A. Cogli L. Alifano P. Bucci C. (2007). The *Neisseria meningitidis* capsule is important for intracellular survival in human cells. *Infect. Immun.* 75 3594–3603. 10.1128/iai.01945-06 17470547 PMC1932921

[B49] Tobiason D. M. Seifert H. S. (2010). Genomic content of *Neisseria* species. *J. Bacteriol.* 192 2160–2168. 10.1128/jb.01593-09 20172999 PMC2849444

[B50] Trauble H. Overath P. (1973). The structure of *Escherichia coli* membranes studied by fluorescence measurements of lipid phase transitions. *Biochim. Biophys. Acta* 307 491–512. 10.1016/0005-2736(73)90296-4 4581497

[B51] Tsutsumi-Ishii Y. Nagaoka I. (2003). Modulation of human beta-defensin-2 transcription in pulmonary epithelial cells by lipopolysaccharide-stimulated mononuclear phagocytes via proinflammatory cytokine production. *J. Immunol.* 170 4226–4236. 10.4049/jimmunol.170.8.4226 12682256

[B52] van Deuren M. Brandtzaeg P. Van Der Meer J. W. (2000). Update on meningococcal disease with emphasis on pathogenesis and clinical management. *Clin. Microbiol. Rev.* 13 144–166. 10.1128/cmr.13.1.144 10627495 PMC88937

[B53] Virji M. (2009). Pathogenic *Neisseriae*: surface modulation, pathogenesis and infection control. *Nat. Rev. Microbiol.* 7 274–286. 10.1038/nrmicro2097 19287450

[B54] Vylkova S. Nayyar N. Li W. Edgerton M. (2007). Human beta-defensins kill Candida albicans in an energy-dependent and salt-sensitive manner without causing membrane disruption. *Antimicrob. Agents Chemother.* 51 154–161. 10.1128/aac.00478-06 17074797 PMC1797696

[B55] Wassing G. M. Bergman P. Lindbom L. Van Der Does A. M. (2015). Complexity of antimicrobial peptide regulation during pathogen-host interactions. *Int. J. Antimicrob. Agents* 45 447–454. 10.1016/j.ijantimicag.2014.11.003 25532742

[B56] Wassing G. M. Ilehag N. Frey J. Jonsson A. B. (2021). Modulation of human beta-defensin 2 expression by pathogenic *Neisseria meningitidis* and Commensal *Lactobacilli*. *Antimicrob. Agents Chemother.* 65 120–135. 10.1128/AAC.02002-20 PMC8097439 33468461

[B57] Wolfgang M. Park H. S. Hayes S. F. Van Putten J. P. Koomey M. (1998). Suppression of an absolute defect in type IV pilus biogenesis by loss-of-function mutations in pilT, a twitching motility gene in *Neisseria gonorrhoeae*. *Proc. Natl. Acad. Sci. U.S.A.* 95 14973–14978. 10.1073/pnas.95.25.14973 9844000 PMC24560

[B58] Woodhams K. L. Benet Z. L. Blonsky S. E. Hackett K. T. Dillard J. P. (2012). Prevalence and detailed mapping of the Gonococcal genetic island in *Neisseria meningitidis*. *J. Bacteriol.* 194 2275–2285. 10.1128/jb.00094-12 22366419 PMC3347088

[B59] Yasir M. Willcox M. D. P. Dutta D. (2018). Action of antimicrobial peptides against bacterial biofilms. *Materials* 11:2468. 10.3390/ma11122468 30563067 PMC6317029

[B60] Zasloff M. (2002). Antimicrobial peptides of multicellular organisms. *Nature* 415 389–395. 10.1038/415389a 11807545

