# Supplementary material for: DNA Blocks the Lethal Effect of Human Beta-Defensin 2 Against Neisseria meningitidis
Source: Front Microbiol. 2021 Jun 30;12:697232. doi: 10.3389/fmicb.2021.697232 (PMC8278289; doi:10.3389/fmicb.2021.697232)
Supplement: Supplementary file 1 [file Data_Sheet_1.PDF]

Supplementary Material

Meningococcal DNA binds to human beta-defensin 2 and blocks its lethal effect against the bacteria

Gabriela M. Wassing[1], Kenny Lidberg[1], Sara Sigurlásdóttir[1,#], Jonas Frey[1], Kristen Schroeder[2], Nathalie Ilehag[1], Ann-Christin Lindås[1], Kristina Jonas[2] and Ann-Beth Jonsson[1*]

- Corresponding author: Ann-Beth Jonsson, E-mail: ann-beth.jonsson@su.se

Figure S1

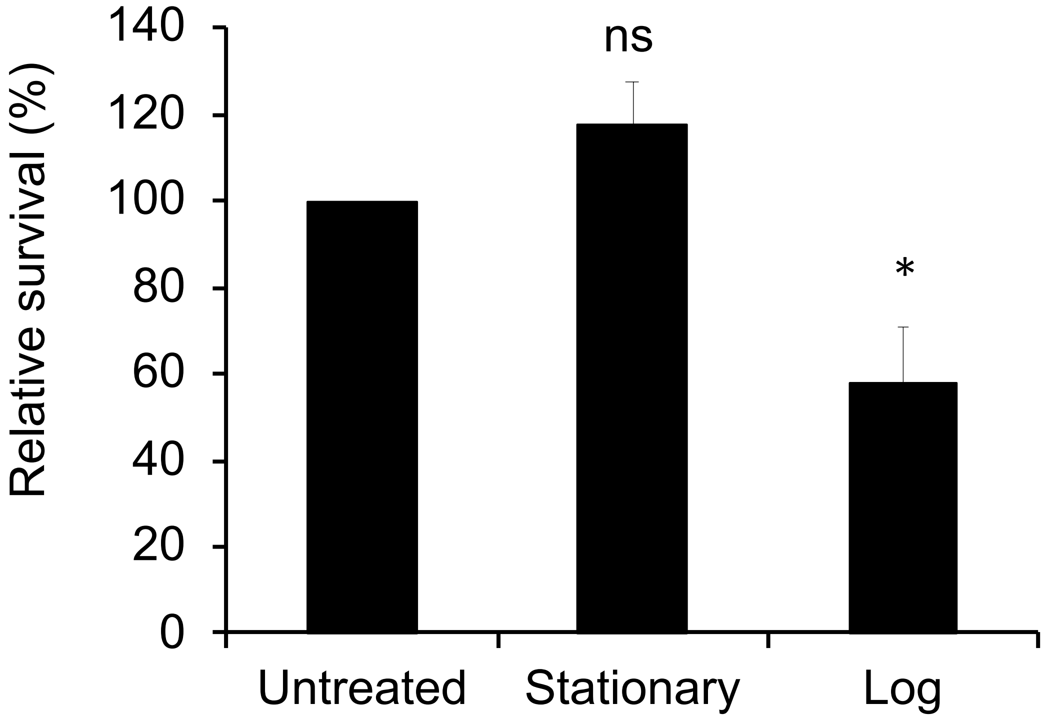

Fig. S1. Survival of bacteria, grown to log phase or stationary phase, incubated with 5 μM hBD2 for 2 h. Viable counts were determined by plating. Survival is expressed relative to the untreated control. Data are represented as the mean survival, with error bars representing the standard deviation. Significance was tested against the untreated control. The assay was performed in triplicate three times. **P*<0.05; ns, nonsignificant.

Figure S2

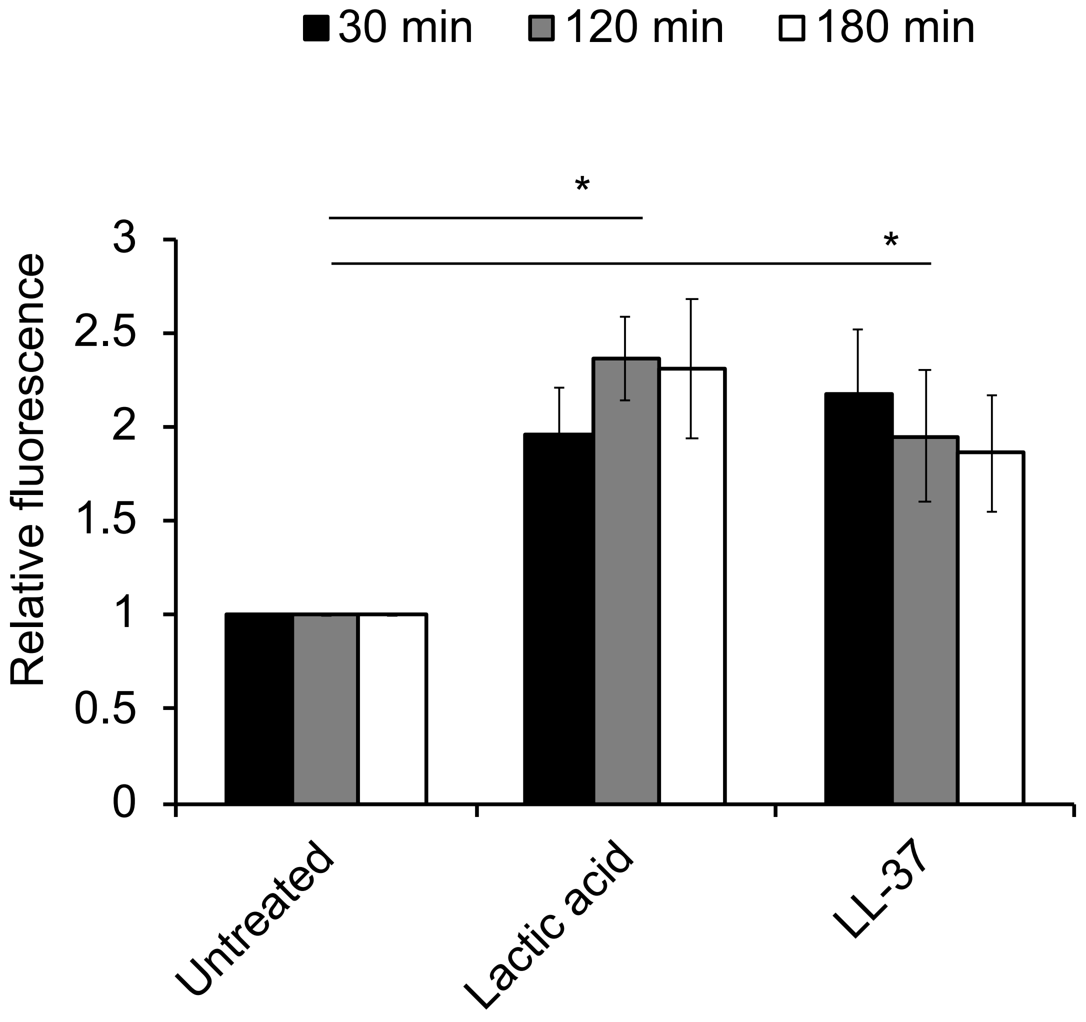

Fig. S2. LL-37 triggers membrane permeability similar to lactic acid in NPN assays.

Outer-membrane permeabilization was assessed by NPN fluorescence. Log-phase bacteria were incubated with NPN (10 µM) and lethal concentrations of LL-37 for 3 h, *i. e.* 10 µM. LL-37 has been shown to kill *N. meningitidis* at concentrations above 5 µM (Jones et al., 2005) Lactic acid was used as a positive control. Background fluorescence (NPN in the absence of bacteria or hBD2) was subtracted. Values are expressed relative to the untreated control. The assay was performed in duplicate at least three times. Data are represented as the mean values, with error bars representing the standard deviation. Significance was tested against the untreated control. *P<0.05.

Figure S3

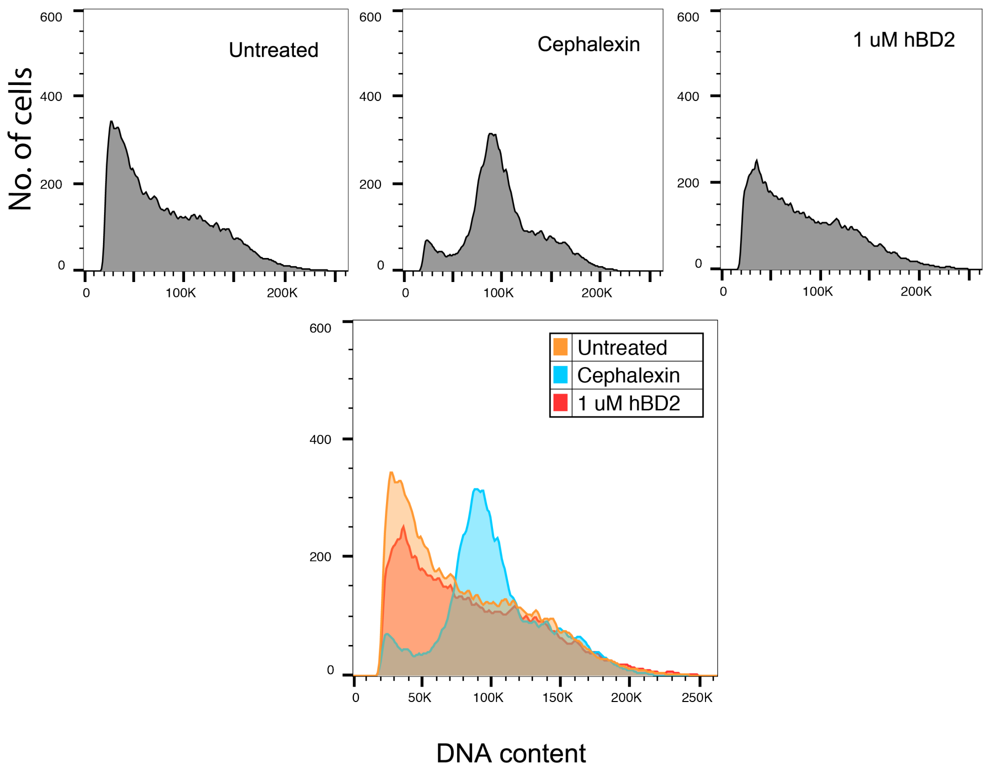

Fig. S3. Flow cytometry analysis of *N. meningitidis* Δ*pilE* in the presence of hBD2. Log-phase bacteria were incubated with 1 μM hBD2 or cephalexin (10 μg/ml) for 2 h. Bacteria were washed in PBS, fixed, permeabilized, and resuspended in sodium citrate buffer. DNA was stained with SYTOX Green, and DNA content was analyzed by flow cytometry. The assay was performed in single samples three times.

Figure S4

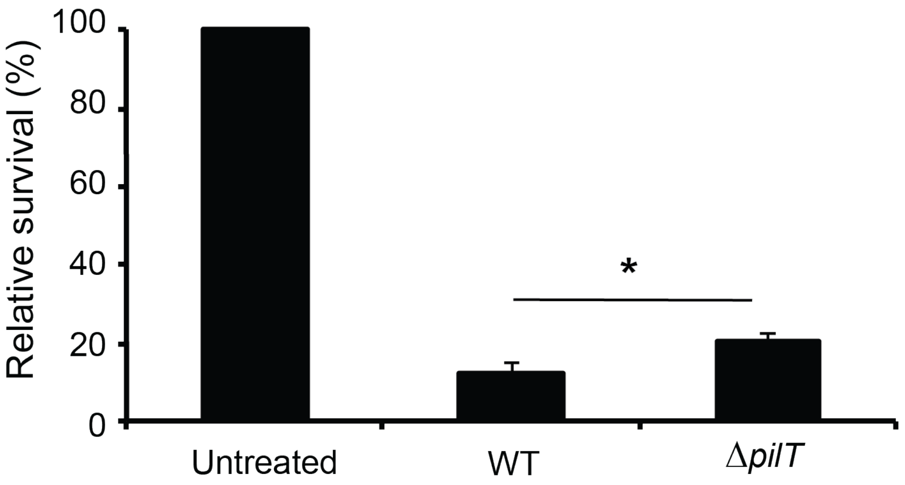

Fig. S4. hBD2-binding and aggregation of wild-type and Δ*pilT* bacteria. Survival of log-phase bacteria of wild-type (WT) and Δ*pilT* incubated with 1 μM hBD2 for 3 h. Viable counts were determined by plating. Values are expressed as the mean survival relative to the untreated control. The assay was performed in triplicate at least three times.
